# Does α-Amino-β-methylaminopropionic Acid (BMAA) Play a Role in Neurodegeneration?

**DOI:** 10.3390/ijerph8093728

**Published:** 2011-09-16

**Authors:** Alexander S. Chiu, Michelle M. Gehringer, Jeffrey H. Welch, Brett A. Neilan

**Affiliations:** The School of Biotechnology and Biomolecular Sciences, The University of New South Wales, Sydney, NSW 2052, Australia; E-Mails: a.chiu@student.unsw.edu.au (A.S.C.); mmgehringer@yahoo.com (M.M.G.); j.welch@unsw.edu.au (J.H.W.)

**Keywords:** BMAA, neuron, glia, neural, neurodegeneration, excitotoxicity, ALS, PDC, cycad, toxicology, cyanobacteria, Alzheimer’s, Parkinson’s

## Abstract

The association of α-amino-β-methylaminopropionic acid (BMAA) with elevated incidence of amyotrophic lateral sclerosis/Parkinson’s disease complex (ALS/PDC) was first identified on the island of Guam. BMAA has been shown to be produced across the cyanobacterial order and its detection has been reported in a variety of aquatic and terrestrial environments worldwide, suggesting that it is ubiquitous. Various *in vivo* studies on rats, mice, chicks and monkeys have shown that it can cause neurodegenerative symptoms such as ataxia and convulsions. Zebrafish research has also shown disruption to neural development after BMAA exposure. *In vitro* studies on mice, rats and leeches have shown that BMAA acts predominantly on motor neurons. Observed increases in the generation of reactive oxygen species (ROS) and Ca^2+^ influx, coupled with disruption to mitochondrial activity and general neuronal death, indicate that the main mode of activity is via excitotoxic mechanisms. The current review pertaining to the neurotoxicity of BMAA clearly demonstrates its ability to adversely affect neural tissues, and implicates it as a potentially significant compound in the aetiology of neurodegenerative disease. When considering the potential adverse health effects upon exposure to this compound, further research to better understand the modes of toxicity of BMAA and the environmental exposure limits is essential.

## 1. The Cycad Hypothesis

Medical research attention was drawn towards Guam in 1953 when it was reported that the incidence of an amyotrophic lateral sclerosis/Parkinson’s disease complex (ALS/PDC) within the local Chamorro people was 100 times higher than the rest of the world [1–3]. After failing to identify any clear genetic correlation to this observation, attention was turned to environmental/cultural factors that might be responsible [2,3]. The use of cycad (*Cycas circinalis*) flour to make tortillas, soups and dumplings by the native Chamorro people [4,5], coupled with various field reports that livestock developed progressive and irreversible ataxia after ingesting cycads [6], led to the suggestion that cycad consumption could be the cause of the human condition [5], and thus the cycad hypothesis was born. In 2007 a group of biostatisticians, led by Borenstein, conducted an in depth population study of the Chamorro people, statistically showing that eating cycads presented the highest associated risk of developing ALS/PDC [7].

## 2. Proving the BMAA Link

**Figure 1:**
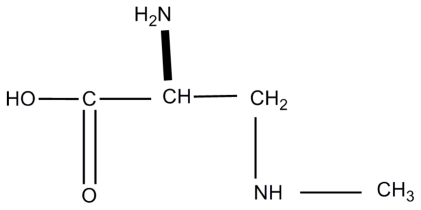
The chemical structure of β-methylaminoalanine (BMAA).

In 1967 Vega and Bell [8] isolated a non-protein amino acid, α-amino-β-methylaminopropionic acid, later renamed BMAA (Figure 1), from cycad seeds. By injecting chemically synthesised BMAA into chicks and rats at 3–7 μmoles/g body weight for chicks, and 6–14 μmoles/g body weight for rats, and subsequently observing weakness, convulsions and general loss of coordination in both animals they determined that BMAA possessed neurotoxic properties [8,9].

Despite this observation, little attention was paid to BMAA until 1987, when Spencer *et al.* correlated elevated incidence of ALS in communities in the Kii peninsula of Japan [10] and Irian Jaya (West New Guinea) [11] to the traditional use of cycad pulp and sap in medicinal broths and in concoctions used to treat wounds. They hypothesised that BMAA was the cycad component that caused ALS and possibly Parkinson’s and Alzheimer’s diseases in Guam and elsewhere [12]. The group then conducted a major experiment in which they fed BMAA (100 to 250 mg/kg) to macaques for up to 12 weeks and observed a variety of symptoms indicating that the animals were suffering from neurodegeneration [13]. This was quickly questioned by Duncan *et al.* [14], who suggested that more than 80% of BMAA was removed from cycad seeds during processing, and by Garruto *et al.* [15] who calculated (based on the knowledge of the time) that the doses used were equivalent to a 70 kg man consuming 1500 kg of cycad flour. These claims led many to believe that BMAA could not possibly be the causative agent, especially when considering the report that at least 85% of the free BMAA was removed from the flour with a single wash, thereby making it impossible to consume toxic quantities [16].

In 2002, Cox and Sacks [17] rejuvenated the idea by proposing a biomagnification process of BMAA accumulation involving flying foxes that fed on cycads. The flying foxes were part of the traditional diet of the Chamorro people, meaning that the concentrations of BMAA actually consumed by humans were much higher than previously thought. Monson *et al.* [18] compiled further evidence correlating increases in flying fox consumption with increases in ALS incidence, thereby providing support for this idea. Whilst the native species of flying foxes are now almost extinct in Guam, testing of dried skin samples from museum specimens revealed BMAA concentrations equivalent (per weight) to up to 1014 kg of processed cycad flour [19], supporting the bioaccumulation hypothesis.

At this point in time the source of BMAA in cycads was unknown. In 2003 Cox *et al.* [20] revealed that BMAA was present in coralloid roots of cycads, but not in roots with normal morphology. It was already known that cyanobacteria lived in the coralloid roots of cycad plants, where they exist as nitrogen fixing symbionts [21]. Cyanobacteria isolated from coralloidal roots were then found to produce BMAA [20]. Subsequent testing of a variety of cyanobacterial species revealed that over 90% of all genera tested, encompassing all five sections of this phylum, produced BMAA [22,23]. The group of Marler *et al.* [24] have suggested that cycad plants can produce BMAA in normal roots lacking symbiotic interaction. While this raises doubts over cyanobacterial involvement, they do suggest that inoculation with cyanobacteria may induce increased production of BMAA by the cycad roots.

In 2003, studies conducted by Banack and Cox [25] showed that, within cycad plants, BMAA was concentrated in the seeds, which are ground up to make flour, that is then washed and used for cooking. Another study conducted by Murch *et al.* [26] detected BMAA in the brain tissues of six out of six Chamorro people who had died of ALS/PDC as well as one asymptomatic Chamorro individual (although at lower concentrations). Interestingly the same study also found BMAA at significant concentrations in the brain tissue of two Canadians who had died of Alzheimer’s disease (AD), suggesting that BMAA may play a role in various types of neurodegenerative disease [26]. These brain tissue samples had been fixed in paraformaldehyde prior to storage in a 15% buffered sucrose maintenance solution [26]. In this, and an associated study, BMAA was detected in protein precipitates, revealing that it can exist in an unknown peptide bound form that had not previously been quantified. The concentrations of this bound form were 10–240 fold higher than those of free BMAA, showing that the accumulated levels were much higher than was previously thought [27]. HPLC and mass spectrometry were used to identify and quantify BMAA in tested samples with a detection limit of 100 pmol [26,27]. These findings were brought into question in 2005 when Montine *et al.* [28] conducted their own study and failed to detect any free BMAA in any brain samples taken from the Chamorro ALS/PDC victims or US AD victims. The same research group again failed to detect free or protein-associated BMAA in similar *post mortem* brain samples in 2009 [29]. These observations led them to conclude that BMAA did not accumulate in brain tissues and therefore could not cause neurodegeneration. The tissue samples used in these studies had been flash frozen in liquid nitrogen and stored at −80 °C, without the use of any fixative or preservative. Both groups prepared their samples using trichloracetic acid (TCA) protein precipitation techniques, however a different HPLC-fluorescence detection (HPLC-FD) method was used by Montine *et al.* [28] with a claimed detection limit of 1 pmol (100 times more sensitive than the method of Murch *et al.* [26]). The different methods used by the group of Montine *et al.* [28,29] have been suggested by some parties [30,31] to be responsible for a lack of detection, rather than an actual lack of BMAA presence in tested samples. The 6-Aminoquinolyl-*N*-hydroxysuccinimidyl carbamate method used by Murch *et al.* [26] is more stable than the other methods [32], and is the preferred method for analysis of amino acids [33]. Instability of the method used by Montine *et al.* might explain the lack of detection.

In 2006 Banack *et al.* [4] tested various organs, including the brain and muscle samples of flying foxes from Guam as well as the nearby islands of Yap and Samoa, finding significant levels of BMAA contained in all Guamanian samples, as well as detectable levels in most samples from Yap. They also proposed that consumption of other cycad foraging animals, such as wild deer and boars, could increase BMAA ingestion in the Chamorros. A study by Pablo *et al*. [34] detected high concentrations of BMAA in 49 out of 50 postmortem brain samples from ALS and AD sufferers in North America, and importantly, no BMAA was detected in healthy controls. This provided further evidence that bioaccumulation of BMAA in neurodegenerative disease sufferers may be a global concern.

In 2007, Banack *et al*. [35] employed five different detection methods to show that laboratory cultures of free-living marine *Nostoc* species produce BMAA, proving that it could be produced globally. The ubiquitous nature of cyanobacteria means that given the right conditions, bioaccumulation of BMAA could potentially occur in any of the greatly varying environments in which cyanobacteria are found [26]. This presumption is supported by the growing number of reports of BMAA detection in different environments. Bioaccumulation of BMAA has been shown in aquatic species such as zooplankton, fish, mussels and oysters in the Baltic Sea [36], as well as in food chains in South Florida [37]. BMAA has also been detected in fresh water lakes in China [38], and in desert dust from the Middle East [39]. The potential for bioaccumulation is supported by the *in vivo* uptake of BMAA by the aquatic macrophyte *Ceratophyllum demersum* in an experimental system [40], and bioconcentration in the zooplankton *Daphnia magna* [41].

## 3. Neurodegeneration is Caused by Excitotoxicity

Excitatory amino acids (EAAs) act as neurotransmitters within the nervous system [42]. Their action is performed by binding to EAA receptors that are present on all nerve cells, particularly concentrated in the synapses. EAA receptors mediate excitatory synaptic transmission via control of the flow of ions, most notably Ca^2+^, K^+^, Na^+^, Mg^2+^ and Cl^−^ [43]. Malfunctions in this system can lead to neurons being damaged and fatally compromised, a process known as excitotoxicity [44]. Excitotoxic cell death involves prolonged depolarization of neurons, changes in intracellular calcium concentrations, and the activation of enzymatic and nuclear mechanisms of cell death [45]. The main EAA receptors are quisqualate/α-amino-3-hydroxy-5-methyl-4-isoxazolepropionic acid (AMPA), *N*-methyl-d-apartate (NMDA) and metabolic glutamate receptors (mGluR), all of which are activated by glutamate and similar substances. A review by Doble [45] explained all these concepts and activities in great detail. The idea that excitotoxicity is a main player in neurodegenerative disease is supported by many studies that have shown that there is an increased level of glutamate found in the cerebrospinal fluid of ALS patients [46–50].

## 4. Summary of the Multiple Mechanisms of BMAA Activity

With substantial and ever growing evidence that BMAA does play a role in the onset and progression of neurodegenerative diseases, the most important question is; what mode of activity does BMAA exert? Although BMAA had not yet been discovered, Dastur [51] fed cycad flour pancakes to Rhesus monkeys in 1964, observing various effects including muscle atrophy and neurodegeneration. Although given that cycad flour was used rather than pure BMAA, these effects may have potentially been influenced by other compounds present in the mixture. Immediately following the discovery of BMAA in 1967, Bell *et al.* [52,53] also conducted some very basic toxicity assays by intraperitoneally injecting BMAA into chicks and rats and observing the development of neurological symptoms via impairment of normal physical function in both cases. These findings were repeated in 1972 by Polsky *et al.* [54], with addition of mice as test subjects. In all cases all the animals suffered the same symptoms, namely weakness, convulsions and general lack of coordination. After this study no productive research using BMAA was conducted until 1987, when the revolutionary investigation of Spencer *et al.* [13] was reported. In those studies macaques were fed 100–350 mg/kg BMAA daily for up to thirteen weeks, resulting in corticomotoneuronal dysfunction, Parkinsonian features and behavioural abnormalities. The 1991 study of Rakonczay *et al.* [55] and 1993 study of Matsuoka *et al.* [56] produced similar findings, with BMAA injected rats displaying acute physical impairment including poor balance, poor coordination and convulsions. Contrary to these observations, the 1989 study of Perry *et al.* [57] fed high doses of BMAA (15.5 g/kg total, 500 mg/kg or 1000 mg/kg doses) to mice over an 11 week period, and observed no behavioural abnormalities during the course of the experiment. Analysis of brain and liver samples collected *post euthanasia* failed to find any evidence of neurochemical or neuropathological changes in the any of the sample animals [57]. Similarly, in 2006 the group of Cruz*-*Aguado *et al.* [58] fed 28 mg/kg of BMAA, which was an exposure level they deemed to be an accurate environmental representation, to mice daily for 30 days. In this study they found no indication that neurological damage had occurred [58]. In the critical review by Karamyan and Speth [31], the authors raise doubts over the methods used by Perry *et al.* [57] to observe behavioural differences, and over the doses used by Cruz*-*Aguado *et al.* [58], as possible explanations for their negative observations. It was also suggested by Banack *et al.* [59] that the mouse model may be a poor model to demonstrate the neurotoxicity of BMAA. It is evident that the bulk of early research was focused on either detecting BMAA in known (deceased) neurodegenerative sufferers, or observing behavioural changes in lab animals fed or injected with BMAA. While this information was useful it did nothing to explain the actual mechanisms of BMAA activity.

**Figure 2:**
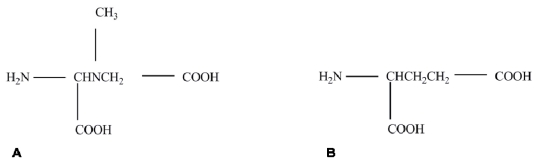
Comparison of the structure of (**A**) β-carbamate (BMAA adduct) and (**B**) glutamtic acid (glutamate).

The first mechanistic BMAA research was performed in 1988 when Weiss and Choi [60] discovered that BMAA only displayed activity *in vitro* when a physiological concentration (10 mM and above) of bicarbonate (HCO_3_^−^) ions were present in the media. This discovery was soon followed by Richter and Mena’s [61] observation that BMAA inhibited glutamate binding in the synaptic junctions of rat brains at 1 mM, but only in the presence of 20–25 mM bicarbonate ions. The observation that effective inhibition of glutamate receptors was not achieved by BMAA at the extremely high level of 10 mM, independent of bicarbonate ions, supported the findings of Weiss and Choi [60], that HCO_3_^−^ was required for BMAA activity to occur. Follow up experiments showed that BMAA could bind to NMDA and non-NMDA receptors on mouse cortical neurons [62]. The dependence of BMAA on HCO_3_^−^ was a critical discovery as it greatly affected the results of experiments conducted using freshly isolated tissues where experimental reagents are generally simple and defined, and often did not contain HCO_3_^−^. Using these leads Myers and Nelson [63] identified a β-carbamate of BMAA (formed in the presence of bicarbonate), that shares structural characteristics with glutamic acid (glutamate, see Figure 2). This led to an explanation of a mechanism of activity, as it suggested that BMAA may have the ability to inhibit glutamate receptors. From this point on all researchers used media and/or buffers supplemented with a minimum of 20 mM bicarbonate in all active *in vitro* assays.

In 1990 Lindström *et al*. [64] gave intracerebral injections (10 or 400 μg) of BMAA to mice and after one week they noticed a decrease in noradrenalin (NA) levels in the hypothalamus, while there was no effect on dopamine or serotonin levels. No physical or behavioural effects were observed in the exposed animals. They suggested that the decrease in NA levels in the tissue may have been the result of BMAA activity on NMDA receptors, causing a release of NA. Copani *et al.* [65] conducted a thorough investigation of BMAA binding capabilities and specificities by performing *in vitro* assays. Brain slices and mixed primary cultures taken from 8-day old rats were exposed to BMAA at 1 mM in conjunction with various neural metabolites and antagonists of NMDA. Their results indicated that BMAA acts as a mixed agonist of metabotropic and NMDA receptors, and as seen in other studies, BMAA activity was enhanced by the presence of bicarbonate ions at 25 mM [65]. The groups of Rakonczay *et al.* [55] and Matsuoka *et al.* [56] performed a series of binding assays using various receptor antagonists after giving intracerebroventricular injections of BMAA (500 μg/day, for up to 60 days) to rats. Their results indicated that BMAA has a mixed agonistic effect on EAA, NMDA and quisqualate/AMPA receptors in the synapse. In 1991–1992 Duncan’s research group conducted a number of experiments relating to the body’s ability to take up BMAA after oral exposure and transport and accumulate it in the brain. When cynomologous monkeys were orally dosed with BMAA, a maximum of 20% of the administered dose was metabolized, and no greater than 2.1% was excreted indicating that approximately 80% of orally consumed BMAA was absorbed into systemic circulation [66]. The 1998 study by Kisby *et al.* [67] reported that BMAA was detected in the cerebrospinal fluid of orally dosed monkeys, and in the brain tissue of intraperitoneally dosed rats, suggesting that BMAA is able to cross the blood-brain barrier. In a later study, Duncan *et al.* [68] demonstrated in rats that, after intravenous injection, acute BMAA levels in the brain peaked at eight h *post administration*. They also demonstrated that BMAA is taken up into the brain by the large neutral amino acid carrier of the blood-brain barrier, which suggests that uptake may be sensitive to the same factors that affect neutral amino acid transport such as diet, metabolism, disease and age [69]. In essence this means that BMAA uptake into the brain may be increased in times of stress.

Brownson *et al.* [70] assayed rat brain cells for changes in the concentration of Ca^2+^ in the presence of BMAA (5 mM) with or without HCO_3_^−^ ions. This experiment indicated that there was a small increase in intracellular Ca^2+^ concentration with BMAA only, but a large increase when BMAA and HCO_3_^−^ were added together. This further supports the belief that BMAA is dependant on HCO_3_^−^ as a cofactor and that the correspnding β-carbamate is the active compound. It also suggests another potential mechanism of activity as impairment to intracellular calcium homeostasis has been shown to cause disruptions in Ca^2+^-dependant cascades that lead to neuronal cell death and neurodiseases [71,72].

The study of Nedeljkov *et al*. [73] measured the membrane input resistance of the nerve cells of the leech *Haemopis sanguisuga* after treatment with BMAA (100 μM–10 mM) and HCO_3_^−^ (20 mM). A significant reduction in input membrane resistance was measured, indicating that BMAA depolarizes the cell by increasing membrane permeability and conductance.

In 2007, Buenz and Howe [74] intracranially injected 10 μL of 100 mM BMAA into mice that were then euthanized at 24 h *post exposure*. This study showed that BMAA caused injury to hippocampal neurons. They also demonstrated that BMAA increasingly caused a degree of cell death in NSC-34 cells (a mouse derived spinal motor neuron-like cell line) as the amount of BMAA administered increased from 100 μM up to 1 mM. A study conducted by Lobner *et al.* in 2007 [75] showed that BMAA at concentrations as low as 10 μM can potentiate neuronal injury caused by other known neurotoxins such as amyloid-β and MPP^+^. This observation holds great significance determining that very low concentrations of BMAA (orders of magnitude lower than previously thought) can potentially cause serious neurological damage if other factors are involved. This study also showed that BMAA has three-fold activity by causing excitotoxicity on NMDA and metabotropic glutamate receptor subtype 5 (mGluR5) receptors, and via oxidative stress. This supports the notion that BMAA may play a role in a variety of different neurodegenerative conditions. Rao *et al.* [76] concluded that low concentrations of BMAA (30 μM) selectively injure motor neurons via excitotoxic activation of AMPA/kainite receptors. They also showed that BMAA induces increases in Ca^2+^ concentrations and the generation of selective reactive oxygen species (ROS) in motor neurons, with minimal effect on other spinal neurons. Liu *et al.* [77] validated the three-fold activity of BMAA described by Lobner *et al.* [75], as well as suggesting that the mechanism BMAA uses to induce oxidative stress is through inhibition of the cystine/glutamate antiporter system Xc^−^, leading to glutathione depletion and oxidative stress [77]. In 2009, Nunn and Ponnusamy [78] found that 2,3-diaminopropionic acid, the dimethylated product of BMAA, and methylamine were formed in liver and kidney preparations from rats exposed to 10 mM BMAA for 24 h *in vitro*. It is worth noting that this product was not formed in brain tissues in this study. This provides evidence of yet another method of toxicity by BMAA, although the test dose is potentially too high for the result to be environmentally significant. Production of methylamine is significant as it has been shown to produce a state of oxidative stress in rats [79]. In 2009, Karlsson *et al.* [80] injected radioactively labelled BMAA into frogs and mice, then euthanized the animals at 30 min, 1 h, 3 h, 24 h and 12 days *post injection*. The results showed that BMAA interacts/binds with melanin, particularly during its synthesis, and increasingly bioaccumulates in melanin and neuromelanin-containing cells over time. The authors proposed that this may provide a link between BMAA and the PDC symptom of pigmentary retinopathy [80]. Also in 2009, Lopicic *et al.* [81] showed that 1 mM BMAA (with 20 mM bicarbonate) causes *in vitro* membrane potential depolarization of leech nerve cells by action on non-NMDA ionotropic glutamate receptors. A concomitant increase in cell membrane input conductance, as well as an increase in Na^+^ activity and a decrease in K^+^ activity was noted. This indicated that, in addition to AMPA/kainite receptors, BMAA could initiate excitotoxicity through the activation of other non-NMDA ionotropic glutamate receptors. In 2009, Santucci *et al.* [82] injected 5–10 nM BMAA into the eyes of mice, that were then euthanized between 4 and 24 h *post administration*. Increases in retinal neuron death and the production of ROS were observed in this study. Also in 2009, Purdie *et al.* [83] exposed zebrafish embryos to BMAA at up to 50,000 μg/L (approx. 300 mu;M) for 5 days. This exposure resulted in a range of neuromuscular and developmental abnormalities, which could be directly related to disruptions to the glutamatergic signalling pathways.

In 2010, Cucchiaroni *et al.* [84] found rat neurons exposed to 1 or 3 mM BMAA displayed increases in the production of ROS, influx of Ca^2+^ and a massive release of cytochrome-c (cyt-c) into the cytosol. This study also demonstrated that activity was predominantly mediated via mGluR1 receptors. These observations indicate disruption to mitochondrial activity, excitotoxicity, and induction of apoptosis induced by exposure to BMAA. More recently Karlsson *et al.* [85] injected 50 and 200 mg/kg BMAA into neonatal rats and found that it inhibited neural development leading to long-term cognitive impairment and supporting the zebrafish data implicating BMAA as a developmental neurotoxin [83]. Most recently, Lee and McGeer [86] exposed three different neuronderived human cancer cell lines to BMAA. Interestingly they observed that BMAA did not cause damage to human neurons and concluded that the hypothesis of BMAA causing neurodegeneration in humans was not tenable [86]. It should however be noted, that the cell lines they used were highly proliferative immortalized cells that differ significantly in physiological characteristics from normal neurons *in vivo*. Summaries of both *in vivo* and *in vitro* investigations into the bioactivity of BMAA are presented in Tables 1 and 2 respectively.

When reviewing BMAA literature it quickly becomes clear that there are large differences in opinion. When Borenstein *et al.* [7] proposed their correlation supporting the role of cycads (potential involvement of BMAA), Steele and McGeer [87] raised doubt over the statistics. When Duncan *et al.* [14] indicated that greater than 80% of BMAA is removed from cycad flour during processing (washing), Cheng and Banack [88] claimed that due to sampling methods, the amount of BMAA detected by Duncan *et al.* [14] has been underestimated by 7- to 30-fold and based on the assumption that BMAA is washed away, another candidate compound such as β-sitosterol β-d-glucoside (BSSG) is suggested to play the same proposed role [89]. Various studies have been conducted to indicate that BSSG does display neurotoxic properties [90,91]. Interestingly despite suggestion that BMAA and BSSG are alternatives for each other, involvement of either or both, would support the same cycad hypothesis.

## 5. A Summary of the Mode of Action of BMAA based on the Current Literature

**Figure 3:**
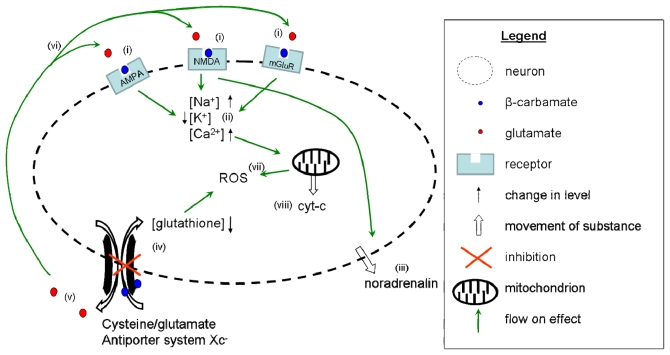
Illustrative summary of the modes of action of BMAA on neurons. *In vivo*, BMAA is present as a β-carbamate (represented by the blue dots), which binds to NMDA, AMPA and mGlu receptors (**i**). Activation of glutamate receptors results in an increase in the levels of Na^+^ and Ca^2+^ in the cell, accompanied by a reduction in K^+^ (**ii**). The cell becomes depolarised and the membrane becomes permeable, as illustrated by the dotted line, and combined with NMDA receptor activity, noradrenalin is released from the cell as a result (**iii**). The cysteine/glutamate antiporter system Xc^−^ is inhibited, as indicated by the red X (**iv**), leading to intracellular depletion of glutathione and an increase in ROS. This inhibition also causes an increase in the release of glutamate (**v**), which then binds to receptors to induce further excitotoxicity (**vi**). All these mechanisms combine to cause an increase in the generation of ROS (**vii**). The elevation of Ca^2+^ leads to overload of the mitochondria resulting in a massive release of cyt-c into the cytosol (**viii**).

The studies listed in Tables 1 and 2, while executed on vastly different test models with varying measurement parameters, can be combined to generate an image of the mechanisms of action of BMAA on a primary motor neuron as illustrated in Figure 3. After being orally consumed, 80% of ingested BMAA passes from the gut into the blood stream [66]. BMAA then crosses the blood-brain barrier via large neutral amino acid carriers [68]. The physiological concentrations of bicarbonate ions (10 mM and above) reacts with BMAA to form a β-carbamate [60]. In this form, BMAA can compete in binding various glutamate receptors, such as NMDA receptors [55,56,64,65], AMPA receptors [55,56], and metabotropic and ionotropic glutamate receptors [65,73,81,84] (Figure 3i). Activation of the various glutamate receptors leads to shifts in cellular ion concentrations resulting in increases in Na^+^ [81] and Ca^2+^ [70,76,84], and a decrease in K^+^ [81] concentrations (Figure 3ii). Activation also causes the cell to become depolarised [73] leading to permeabilisation of the cell membrane, resulting in the release of noradrenalin [64] (Figure 3iii). BMAA also inhibits the cysteine/glutamate antiporter system Xc^−^ [77] (Figure 3iv), preventing the uptake of cysteine, resulting in glutathione depletion, which contributes to increases in oxidative stress. At the same time the system Xc^−^ increases the release of glutamate from the cell (Figure 3v), which can then bind to glutamate receptors increasing damage by excitotoxicity [77] (Figure 3vi). Increases in intracellular Ca^2+^ concentrations disrupt normal mitochondrial function leading to the release of ROS into the cytoplasm, thereby contributing to the observed increases in ROS [75,76,82,84] (Figure 3vii). In addition, cytochrome-c is released from the mitochondria [84] (Figure 3viii) resulting in the induction of apoptosis.

## 6. Concluding Remarks

Whilst the incidence of ALS-PDC on Guam was 100 times that of the world average, it peaked at 120 cases per 100,000 people, meaning that the majority of individuals thought to be exposed to BMAA still escaped disease. Clearly there is still much to learn about the role(s) that BMAA plays in neurodegeneration. Karamyan and Speth have reviewed the available literature on the evidence for and against the involvement of BMAA in the development of ALS/PDC [31]. They concluded that the majority of studies indicate that BMAA is toxic. It is worth noting that the two studies [57,58] that observed no effect both utilized oral administration methods, perhaps implying reduced toxicity via this delivery method. One must also consider that the severe effects observed by Spencer *et al.* [13] were also obtained with oral dosing.

When considering all the published data, it appears certain that BMAA can contribute to the onset and progression of neurodegenerative disease in certain susceptible individuals. It would be useful to focus on better understanding the proposed mechanisms of BMAA activity, as well as identifying new as yet undescribed mechanisms that might play an important role in the overall potency of BMAA. Without a sound understanding of how BMAA truly works, it is impossible to predict the level of risk it poses with any significant degree of confidence. One question posed in the review by Karamyan and Speth [31] that is likely to be answered in the affirmative was “are there interactions between BMAA and other exogenous substances with possible synergetic toxicity?”. The potential dangers of BMAA acting as an accessory or combinatorial toxin, rather than being highly toxic as a sole entity, were indicated by Lobner *et al.* [75] when they demonstrated that BMAA can potentiate the activity of other insults. As BMAA has been shown to be co-present with other cyanotoxins, such as microcystin, anatoxin-a, nodularin and saxitoxin [92], this potentiation capability, may implicate BMAA as an important factor when considering the management strategies of these other toxins. The debate between BSSG and BMAA appears to be very polarized, with acceptance of one causative agent completely ruling out the significance of the other. It may however be more logical to consider the idea that as the two compounds were isolated from the same source, they are likely to be present together environmentally, and could therefore act in a combination, potentially far more potent than either agent alone, to induce neurological damage. There is little doubt that if present in sufficient concentrations, BMAA exerts multiple modes of neurotoxic activity, with perhaps further modes yet to be defined. With growing reports of its presence in varied environments it is important that research to understand the complete nature of BMAA toxicity continue. Equipped with a greater knowledge and understanding of the mechanisms of BMAA toxicity, we will be able to more accurately evaluate and assess the human health risks posed by exposure to this cyanotoxin.

## Figures and Tables

**Table 1:** A chronological summary of mechanisms of BMAA activity determined by *in vivo* research.

Routez of exposure	Species	Dose level, exposure time	Research group and date	Observations
Intraperitoneal injections	Rat Chicken	6–14 μmoles/g body weight 3–7 μmoles/g body weight	Vega and Bell. 1967	Weakness, convulsions and uncoordination
Intraperitoneal injections	Rat Chicken Mouse	6–14 μmoles/g body weight 3–7 μmoles/g body weight 6–14 μmoles/g body weight	Polski *et al.* 1972	Weakness, convulsions and uncoordination
Perorally Intraperitoneal injections	Monkey Rat	100–350 mg/kg, 12 months 500 mg/kg daily, 14 days	Kisby *et al.* 1988	BMAA can cross from gut to blood BMAA can cross the blood brain barrier
Gavage	Monkey	100–350 mg/kg daily, up to 10 weeks	Spencer *et al.* 1987	Corticomotoneuronal dysfunction, Parkinsonian features and behavioural abnormalities
Gavage	Cynomologous monkey	500 mg/kg daily, 18 days, then 500 mg/kg 2 daily, 28 days, then 100mg/kg 2 daily, 30 days	Perry *et al.* 1989	No behavioral or physiological effects observed
Intracerebral injections	Rat	10 μg or 400 μg/150–200 g rat	Lindström *et al.* 1990	Activation of NMDA receptor, release noradrenalin from cells
Intracerebroventricular injections	Rat	500 μg/day 200–250 g body weight, 10–60 days	Rakonczay *et al. 1990* Matsuoka *et al.* 1993	Agonistic effects on NMDA, EAA and AMPA receptors in synapse Physical impairment. Mixed agonistic receptor activity
Gavage and intravenous injections	Cynomologous monkey, rat	2 mg/kg gavage; 1 mg/kg iv 100 mg/kg gavage; 24–400 mg/kg iv	Duncan *et al.* 1991–1992	80% of ingested BMAA enters systemic circulation. BMAA can cross the blood brain barrier. BMAA is transported by neutral amino acid carriers so uptake can be influenced by diet, metabolism, disease and age
Dosed feed pellets	Mouse	28 mg/kg daily, 30 days	Cruz-Aguado *et al.* 2006	No motor, cognitive or neuropathological effect observed
Intracranial injections	Mouse	10 μL of 100 mM, 24 h	Buenz and Howe. 2007	Injury to hippocampal neurons
Intravenous and subcutaneous injections	Mouse and frog	7.3 μg/kg, 30 min, 1 h, 3 h, 24 h, 12 days	Karlsson *et al.* 2009	BMAA interacts/binds melanin, particularly during synthesis, and accumulates in melanin and neuromelanin containing cells increasingly over time
Ocular injections	Mouse	5–10 nmol, 4, 8 and 24 h	Santucci *et al.* 2009	Retinal neuron death and production of ROS

**Table 2:** A chronological summary of mechanisms of BMAA activity determined by *in vitro* research.

Experimental model	Species	Dose level, exposure time	Research group and date	Conclusion
Primary cortical neurons	Mouse	3 mM, 1 h With and without 10–24 mM HCO_3_^−^	Weiss and Choi, 1988	BMAA activity is dependent on bicarbonate at a min. of 20mM
Primary cortical neurons	Mouse	300 μM–3 mM, 24 h	Weiss *et al.* 1989	BMAA has activity on NMDA and non-NMDA receptors
Primary cortical neurons	Rat	1 mM	Richter and Mena, 1989	Inhibition of glutamate binding in synapse, impaired neuron function
Chemical assay		-	Myers and Nelson, 1990	Formation of bicarbonate adduct with structural similarity to glutamate
Brain slices	Rat	1 mM, acute	Copani *et al.* 1991	BMAA acts as a mixed agonist of metabotropic and NMDA receptors
Minced brain	Rat	5 mM, acute	Brownson *et al.* 2002	Impairment of intracellular calcium ion homeostasis. Possible neuronal death. Effects on calcium dependent cascades
Primary nerve cells	Leech	1–10 mM, acute	Nedeljkov *et al.* 2005	Depolarisation of cell, impaired nerve function. Membrane permeabilisation. Activity via glutamate receptors
Primary embryonic spinal cord culture	Mouse	30–1000 μM, 20–24 h	Rao *et al.* 2006	Increase on calcium ion concentration and ROS. Selective damage to motor neurons
Primary mixed cortical cells	Mouse	0.1–10 mM, 24 h 3 mM, 3 h (DCFDA)	Lobner et al. 2007	Potentiation of other insults, makes cells more sensitive to other compounds. Increase in ROS
NSC-34 cells	Mouse	50–1000 μM, 18 h	Buenz and Howe 2007	Dose dependent death of NSC-34 cells
Primary mixed cortical cell cultures	Mouse	3 mM, 3 h	Liu *et al.* 2009	Induction of oxidative stress is through inhibition of the cystine/glutamate antiporter system Xc^−^
Brain slices. Brain, liver, kidney homogenates	Rat	10 mM, 30 min for slices 1 h for homogenates	Nunn and Ponnusamy, 2009	The dimethylated product of BMAA, 2,3-diaminopropionic acid was formed in liver and kidney (but not brain) preparations
Nerve cells	Leech	100–3000 μM, acute	Lopicic *et al.* 2009	Action on non-NMDA ionotropic glutamate receptors, with a concomitant increase in cell membrane input conductance, as well as an increase in Na^+^ activity and a decrease in K^+^ activity. Possible initiation of excitotoxicity through activation of non-NMDA ionotropic glutamate receptors
Brain slices	Rat	100–10000 μM, acute	Cucchiaroni *et al.* 2010	BMAA activates mGluR1 receptors to cause neuronal degeneration Massive release of cyt-c into cytosol
